# 99 Burn Center Nurse Case Peer Review

**DOI:** 10.1093/jbcr/iraf019.099

**Published:** 2025-04-01

**Authors:** Sarah Bryan, Theresa Latacki, Tiffany Ciminello, Anjay Khandelwal

**Affiliations:** Akron Children’s Hospital Burn Center; Akron Children’s Hospital Burn Center; Akron Children’s Hospital Burn Center; Adult and Pediatric Burn Institute at Akron Children’s

## Abstract

**Introduction:**

Peer review is an important practice in medicine to improve quality of care and system processes. This practice typically applies to physicians and is mandated by the American Burn Association for verification. Nursing care is vital to our patient outcomes and can be reviewed in a similar manner. The ANA’s Code of Ethics recognizes that effective peer review is imperative for holding nursing practice to the highest standards. Peer review helps address the boundaries of duty for all nurses including the responsibility to safety, competence and professional growth. Therefore, a nurse case peer review process was created in our burn unit. The NCPR participants review and evaluate individual nursing care events and formulate recommendations regarding each situation based on evidence-based practice and due diligence.

**Methods:**

We practice in a regionally verified adult and pediatric burn center. Our burn department consists of 28 RNs and 2 LPNs between the inpatient unit and outpatient clinic. Nurses in our burn unit are invited to meet once a month to review 1-2 cases and identify any opportunities for performance improvement. RN manager and educators are present as resources during discussion. General triggers for our nurse peer review were established to include code blues, MRT, medication errors, PIV infiltrates, significant falls, HAPI, CAUTI, CLABSI, referrals from multidisciplinary team, safety event referrals, and self-referrals. Tracking and documentation of the NCPR is included in our hospital’s safety event reporting templates and software.

**Results:**

A monthly nurse case peer review meeting was initiated in August of 2024. By September 2024, we have reviewed 2 cases. One case was reviewed after CLABSI trigger, in this case clinical issues were identified, hospital policy was revised and a new clinical product was incorporated. A second case was reviewed after self-referral, in this case clinical practice issues were identified, hospital policy was revised, and our burn dressing change procedure was modified. Changes implemented as a result of NCPR are reported to the multidisciplinary team during PI/QA meetings. By April 2025, we are projected to have reviewed 14 cases based on the indicated triggers.

**Conclusions:**

NCPR is important to provide oversight and review of bedside clinical nursing care to support safe, ethical, and autonomous nursing practice in burn care. Creating and independent NCPR process in our burn unit allows us to foster a culture of patient safety, nursing practice accountability and continuous learning.

**Applicability of Research to Practice:**

Nurse Peer review is the best way to promote practice accountability in nursing. Through the NCPR process, nursing staff ensures compliance with adequate charting and that care is being performed following hospital policies. We ensure quality of nursing care through safe deliverance of standards of care and newly discovered evidence-based practice.

**Funding for the Study:**

N/A

